# Assessment of the therapeutic potential of Hsp70 activator against prion diseases using *in vitro* and *in vivo* models

**DOI:** 10.3389/fcell.2024.1411529

**Published:** 2024-07-22

**Authors:** Mohammed Zayed, Yong-Chan Kim, Byung-Hoon Jeong

**Affiliations:** ^1^ Korea Zoonosis Research Institute, Jeonbuk National University, Iksan, Republic of Korea; ^2^ Department of Bioactive Material Sciences, Institute for Molecular Biology and Genetics, Jeonbuk National University, Jeonju, Republic of Korea; ^3^ Department of Surgery, College of Veterinary Medicine, South Valley University, Qena, Egypt; ^4^ Department of Biological Sciences, Andong National University, Andong, Republic of Korea

**Keywords:** SW02, scrapie, neurodegenerative diseases, heat shock protein 70, ATPase activity, drug

## Abstract

**Introduction:**

Prion diseases are deadly neurodegenerative disorders in both animals and humans, causing the destruction of neural tissue and inducing behavioral manifestations. Heat shock proteins (Hsps), act as molecular chaperones by supporting the appropriate folding of proteins and eliminating the misfolded proteins as well as playing a vital role in cell signaling transduction, cell cycle, and apoptosis control. SW02 is a potent activator of Hsp 70 kDa (Hsp70).

**Methods:**

In the current study, the protective effects of SW02 against prion protein 106-126 (PrP^106-126^)-induced neurotoxicity in human neuroblastoma cells (SH-SY5Y) were investigated. In addition, the therapeutic effects of SW02 in ME7 scrapie-infected mice were evaluated.

**Results:**

The results showed that SW02 treatment significantly increased Hsp70 mRNA expression levels and Hsp70 ATPase activity (*p* < 0.01). SW02 also significantly inhibited cytotoxicity and apoptosis induced by PrP^106-126^ (*p* < 0.01) and promoted neurite extension. *In vivo*, intraperitoneal administration of SW02 did not show a statistically significant difference in survival time (*p* = 0.16); however, the SW02-treated group exhibited a longer survival time of 223.6 ± 6.0 days compared with the untreated control group survival time of 217.6 ± 5.4 days. In addition, SW02 reduced the PrP^Sc^ accumulation in ME7 scrapie-infected mice at 5 months post-injection (*p* < 0.05). A significant difference was not observed in GFAP expression, an astrocyte marker, between the treated and untreated groups.

**Conclusion:**

In conclusion, the potential therapeutic role of the Hsp70 activator SW02 was determined in the present study and may be a novel and effective drug to mitigate the pathologies of prion diseases and other neurodegenerative diseases. Further studies using a combination of two pharmacological activators of Hsp70 are required to maximize the effectiveness of each intervention.

## 1 Introduction

Prion diseases are deadly neurodegenerative diseases that cause neuron damage and motor paralysis (Ritchie and Ironside, 2017). Due to their ability to be transmitted from one host to another, these diseases are referred to as transmissible spongiform encephalopathy (TSE) (Liberski and Ironside, 2015; Ritchie and Ironside, 2017), resulting in the characteristic appearance of giant vacuoles in the brain tissue. In prion diseases, the cellular prion protein (PrP^C^) misfolds into a pathological form (PrP^Sc^), accumulating into insoluble amyloid fibrils in the brain tissue (Caughey, 2003; Kovacs and Budka, 2009). Consequently, prion diseases have been categorized into a group of nervous system disorders known as protein misfolding disorders, including Alzheimer’s disease, Parkinson’s disease, and other neurodegenerative disorders (Scheckel and Aguzzi, 2018). In prion diseases, behavioral disorders, and neuron loss are critically linked to the deposition of misfolded proteins in the brain (Moore et al., 2009). PrP^Sc^ causes neurotoxicity and neurodegeneration (Halliday et al., 2014; Glatzel and Sigurdson, 2019), rendering therapies aimed at restoring proper protein folding and promoting neuroregeneration potentially beneficial. Unfortunately, therapies are not currently available to slow the development of prion disorders (Zayed et al., 2023).

According to current knowledge, different potential prospects for intervention in prion diseases exist including targeting cellular PrPs, inhibiting the conversion of PrP^C^ into PrP^Sc^, and clearing accumulated PrP^Sc^ or blocking its toxicity (Krance et al., 2020). In our previous study, clonidine treatment of prion-infected mice was shown to result in significant clearance of deposited PrP^Sc^ by activating the glymphatic system, the brain’s perivascular waste-clearing mechanism (Kim et al., 2021). However, clonidine did not fully clear all the deposited PrP^Sc^ in the prion-infected mice.

Heat shock proteins (Hsps) are a wide family of molecular chaperones that play critical functions in protein translocation across membranes, refolding, and degradation (Vabulas et al., 2010; Miller and Fort, 2018; Rosenzweig et al., 2019). Hsp 70 kDa (Hsp70) has been shown to regulate protein refolding by utilizing the ATP hydrolysis mechanism (Mayer and Bukau, 2005; Rutledge et al., 2022). In studies of chaperone-assisted disintegration, the importance of Hsps in protecting folding intermediates from aggregation and efficiently clearing misfolded species that affect cell survival was demonstrated (Rutledge et al., 2022). The Hsp70 chaperones are fundamental to the appropriate folding of proteins within cells (Rosenzweig et al., 2019). In genetic studies in which protein misfolding diseases were investigated, a strong association between Hsp70 and disease development was found (Petrucelli et al., 2004). Therefore, Hsp70 has recently emerged as a therapeutic target in neurodegeneration through functionally linked ATP-dependent chaperones (Miller and Fort, 2018). For example, molecular chaperones, specifically Hsp70, have been shown to regulate tau ubiquitination, degradation, and aggregation in neurological diseases such as Alzheimer’s disease (Petrucelli et al., 2004; Jinwal et al., 2009).

Hsps play significant roles in cell signaling transduction, cell cycle, and apoptosis in addition to chaperone activities (Hu et al., 2022). Furthermore, Hsps may promote or hamper neurodevelopment via mechanisms that regulate cell differentiation, neurite outgrowth, cell migration, or angiogenesis (Miller and Fort, 2018; Santana et al., 2020; Dutta et al., 2021). Hsp70 was shown to have protective effects against cardiomyopathies (Marber et al., 1995) and enhanced neuroregeneration in a mouse model for spinocerebellar ataxia type 1 (Cummings et al., 2001). In prion diseases, Hsp70 is highly expressed in the brain tissues of Creutzfeldt-Jakob disease patients (Kovács et al., 2001) and scrapie-infected mice (Kenward et al., 1994), indicating its important role in fibril fragmentation and prion propagation (Chernova et al., 2017). Therefore, Mays et al. showed that Hsp70 reduces toxicity and slows prion disease development, presenting opportunities for therapeutic intervention (Mays et al., 2019). Modulating Hsps and their ATPase activity may be an attractive therapeutic approach for neurodegenerative diseases associated with chaperone activities (Beretta and Shala, 2022; Rutledge et al., 2022). Therefore, exploring a novel approach toward chaperone-based treatments for prion diseases is necessary.

SW02, a dimethylformamide compound, is a specific activator/agonist of Hsp70 and Hsp70 ATPase activity (Evans et al., 2006; Jinwal et al., 2009). SW02 indirectly promotes anti-aggregation activity through Hsp70 in Amyloid-β peptide (Aβ) aggregation (Evans et al., 2006). It has been shown that SW02 increases Hsp70 function by approximately 45% (Jinwal et al., 2009). Therefore, Jinwal et al. reported that modulators of ATPase activity can prevent aggregation of Aβ peptide and may be used to target and modify the pathologies of prion diseases and other tauopathies (Jinwal et al., 2010).

The neuropeptide PrP^106-126^ (106-KTNMKHMAGAAA AGAVVGGLG-126) shares several physicochemical and biological properties with PrP^Sc^, including neurotoxicity, proteinase-K resistance, β-sheet structure, and induction of neuronal cell death (Li et al., 2018). Therefore, it serves as a valuable tool for studying pathophysiology and identifying potential targets for therapeutic intervention in prion diseases. In the present study, the protective effects of the Hsp70 activator (SW02) against PrP^106-126^-induced neurotoxicity in the human neuroblastoma cell line SH-SY5Y were investigated. In addition, the therapeutic potential of SW02 based on survival analysis was explored and the PrP^Sc^ accumulation in ME7 scrapie-infected mice was assessed.

## 2 Materials and methods

### 2.1 Cell culture and reagents

SH-SY5Y were obtained from a Korean cell line bank (Seoul, Korea) and cultured in Dulbecco’s Modified Eagle Medium/Nutrient Mixture F-12 (DMEM-F12; Gibco, Grand Island, NY, United States) supplemented with 10% fetal bovine serum (FBS; Gibco) and 1% antibiotic cocktail (Gibco) at 37°C with 5% CO_2_ in a humidified incubator. PrP^106-126^ (KTNMKHMAGAAAAGAVVGGLG; > 95% purity) was synthesized by Peptron (Yuseong-gu, Daejeon, South Korea). The peptide was dissolved in dimethyl sulfoxide (DMSO) (Biosesang, Gyeonggi-do, Korea) to a concentration of 10 mM and stored at −80°C. The Hsp70 activator, SW02, (Sigma-Aldrich, St. Louis, MO, United States) was also dissolved in DMSO and stored as a 10 mM stock solution at −80°C. All procedures were performed under sterile conditions.

### 2.2 Cell viability assay

To assess the cytotoxicity of the PrP^106-126^ peptide, SH-SY5Y cells were treated with different concentrations (0, 100, and 200 μM) of the peptide for 24 h as described in previously published research (Park et al., 2012; Han et al., 2019). The optimization of concentration and exposure time of PrP^106-126^ was evaluated (Supplementary Figure S1). To assess the effects of SW02, different concentrations of SW02 (0, 1, 5, 10, and 20 μM) were applied to SH-SY5Y cells for 24 h. To evaluate the protective effects of SW02 against the PrP^106-126^ peptide, cells were pretreated with SW02 for 3 h before adding PrP^106-126^ followed by incubation for 24 h. The Cell Counting Kit-8 assay kit (Sigma-Aldrich) was used to evaluate cell viability. The CCK-8 solution was directly added to the cell culture medium and incubated for 2 h at 37°C in 5% CO_2_. A microplate reader (SpectraMax Plus 384, Molecular Devices, California, United States) was used to measure absorbance at 450 nm with a background control sample serving as the blank.

### 2.3 Hsp70 ATPase activity

To examine the Hsp70 ATPase activity of SW02, SH-SY5Y cells were pretreated with SW02 for 3 h followed by PrP^106-126^ for 24 h. Nontreated and SW02 only were used as a control. Cell lysates were then collected, and the Hsp70 ATPase activity was measured using the HSP70 Assay Kit (BPS Bioscience, San Diego, CA, United States) according to the manufacturer’s protocol. In brief, cell lysates were added to a 96-well plate (SPL Life Sciences, Gyeonggi-do, Korea) and mixed with Hsp70 followed by incubation for 30 min at room temperature. The reaction was initiated by adding 50 μM ATP and incubated at 30°C for 60 min before adding ADP-Glo reagent (Promega, #V6930). Luminescence was measured with a multimode plate reader (Perkin Elmer, Massachusetts, United States).

### 2.4 RNA isolation and quantitative real-time polymerase chain reaction

RNA was isolated using TRIzol (Invitrogen), and reverse transcription was performed to convert 1 μg RNA to cDNA using ReverTra Ace-α (Toyobo, Tokyo, Japan). Quantitative real-time polymerase chain reaction (RT-PCR) analysis was performed using SYBR Green dye (Yuseong-gu, Daejeon, Republic Korea). The relative mRNA expression levels were normalized to β-actin. The primer sequences for RT-PCR were as follows: Hsp70: forward: 5′- TGT​GTC​TGC​TTG​GTA​GGA​ATG​GTG​GTA-3′ and reverse: 5′- TTA​CCC​GTC​CCC​GAT​TTG​AAG​AAC-3′, β-actin: forward: 5′- TGG​CAC​CCA​GCA​CAA​TGA​A-3′ and reverse: 5′ CTA​AGT​CAT​AGT​CCG​CCT​AGA​AGC​A-3′.

### 2.5 Anti-apoptotic activity of SW02

Terminal deoxynucleotidyl transferase dUTP end labeling (TUNEL) staining was used to assess the apoptotic cell death according to the manufacturer’s instructions (Abcam TUNEL Assay Kit -HRP-DAB, Abcam, Manheim, Germany). Briefly, SH-SY5Y cells were grown on poly-d-lysine coated 12-well plates at a density of 1 × 10^5^ cells per well. The cells were exposed to SW02 followed by PrP^106-126^ for 24 h. The apoptotic cells were stained in a dark brown color by adding DAB and a phase contrast microscope (Carl Zeiss, Oberkochen, Germany) was used to observe the cells.

Caspase-3 is a prevalent trigger of apoptosis. To examine the anti-apoptotic activity of SW02, SH-SY5Y cells were pretreated with SW02 for 3 h followed by PrP^106-126^ for 24 h. Cell lysates were then collected, and caspase-3 activity was determined using the Caspase-3/CPP32 Colorimetric Assay Kit (BioVision, Waltham, MA, United States) according to the manufacturer’s protocol. In addition, the protein level of caspase-3 has been evaluated (Western blotting section).

### 2.6 Neural extension assay

Retinoic acid (RA, Sigma-Aldrich, St Louis, MO) is a vitamin A derivative and the most well-characterized method for inducing neurite extension in SH-SY5Y cells. To quantify neurite extension, SW02 was applied to SH-SY5Y cells for 24 h. To determine the total length of neurite extension, five microscopic fields were randomly selected. ImageJ software (version 1.52, imagej.nih.gov) was used to measure the mean neurite length under an inverted microscope. The measured neurite lengths from five fields are averaged and represented on the *Y*-axis. At least three experiments were performed, and their mean values were calculated. The experiment was performed with 10 µM RA as a positive control.

### 2.7 Animal experiments

Nara Biotech (Pyeongtaek, Korea) provided C57BL/6J mice for animal experiments. Jeonbuk National University’s Institute of Animal Care and Use Committee (JBNU 2020-080) authorized all experimental protocols. To establish an experimental model for prion disease, C57BL/6 mice (6-week-old) were intraperitoneally inoculated with 100 μL of 1% (w/v) brain homogenate prepared from terminally ill ME7 scrapie-infected mice. One-week post-injection, 100 μL SW02 (50 μg/kg) was administered weekly to assess the therapeutic effects of SW02 against prion infection. ME7 scrapie-infected control mice did not receive any treatment. For a negative control, 100 μL of phosphate-buffered saline (PBS) was administered for 1 week after injection. Mice (n = 3) from the three groups were euthanized 5 months post-injection for proteinase K-resistant PrP^Sc^ accumulation analysis using western blotting. To perform the survival analysis, the mice were monitored daily until neurologic signs developed and then euthanized at the terminal stage. Western blotting analysis was used to assess PrP^Sc^ in brain tissue. To detect PrP^Sc^ in the brain, 50 μg/mL proteinase K was added to quantified samples for 1 h at 37°C. The proteinase K-treated samples were heated to 95°C for 10 min in 5X sample buffer (Thermo Fisher Scientific, Waltham, MA, United States) and the PrP^Sc^ bands were detected using western blotting.

### 2.8 Western blotting

A 10% volume of RIPA lysis buffer (Thermo Fisher Scientific) with a protease inhibitor cocktail (Roche, Munich, Germany) was used to homogenize the whole brain. The homogenized samples were loaded into 12% sodium dodecyl sulfate (SDS) gel lanes. An electrophoretic transfer system (BioRad, Hércules, CA, United States) was used to transfer the proteins to a nitrocellulose membrane (Amersham, Little Chalfont, United Kingdom) for 1.5 h at 100 V. After washing, membranes were blocked for 2 h at room temperature with 5% skimmed milk and then incubated with primary antibodies against the PrP monoclonal antibody (SAF84, Bertin, Montigny le Bretonneux, France), glial fibrillary acidic protein (GFAP) (2E1; Santa Cruz Biotechnology, Dallas, TX, United States), and caspase-3 (#9662; Cell Signaling, MA, United States). After washing, the membranes were incubated for 1 h with horseradish peroxidase-conjugated secondary antibodies (Sigma-Aldrich). Protein levels were normalized to β-Actin (Santa Cruz Biotechnology). A Pierce ECL kit (Thermo Fisher Scientific) was used to detect the targeted proteins. Densitometry was used to quantify the intensity of the signal acquired for each protein using ImageJ software (version 1.52).

### 2.9 Statistical analysis

All data are presented as means ± standard deviation (SD). Statistical analysis was performed using SPSS 25.0 (IBM, Armonk, NY, United States) and the *t*-test or one-way analysis of variance (ANOVA) with the Tukey Comparison Test as a post-test. The symbols *, **, and *** indicate *p* < 0.05, *p* < 0.01, and *p* < 0.001 respectively.

## 3 Results

### 3.1 Cytotoxic effects of PrP^106-126^ and SW02 on SH-SY5Y cell line

**FIGURE 1:**
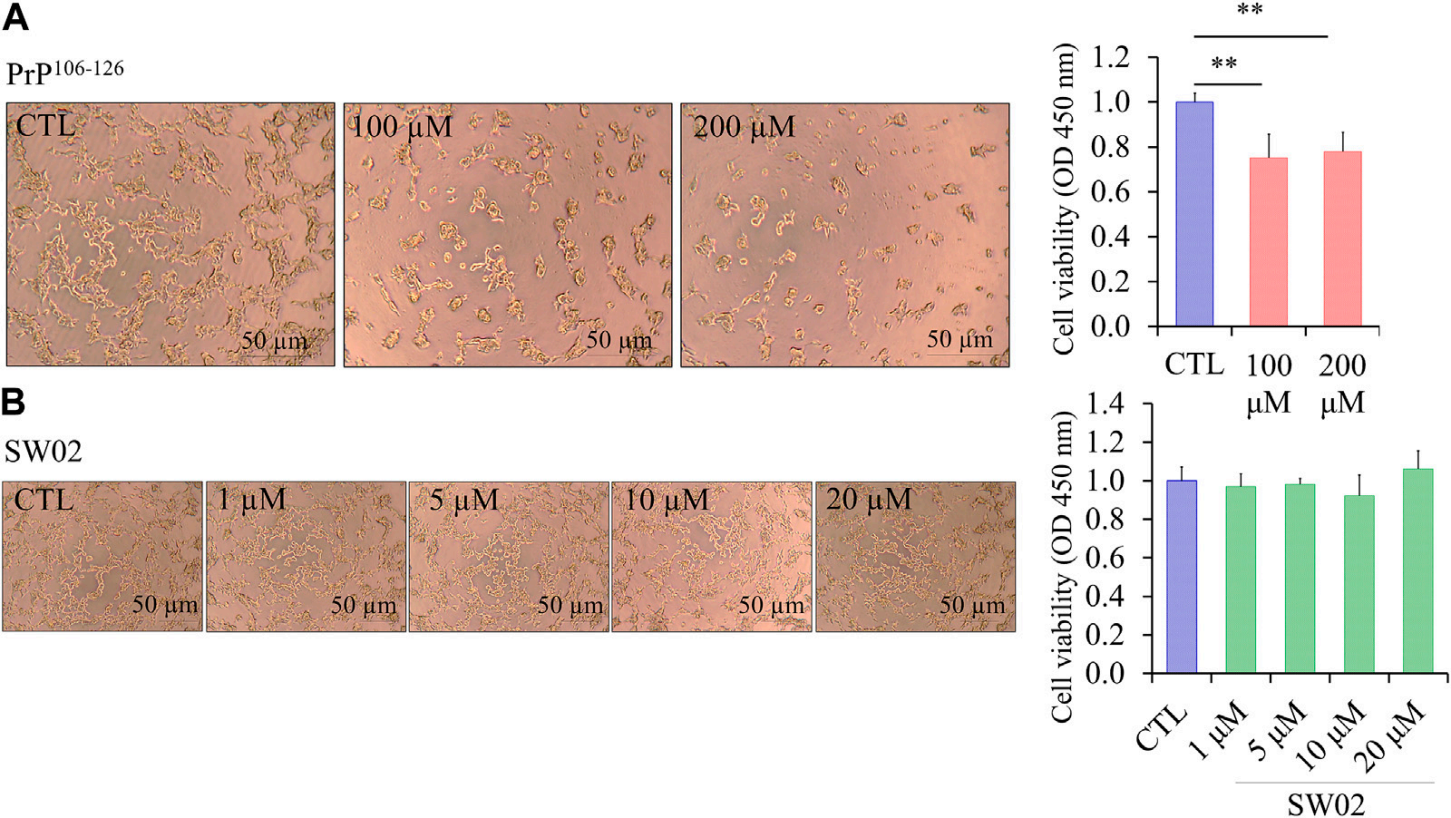
Effect of SW02 and PrP^106-126^ on cellular viability in human SH-SY5Y cells. **(A)** Representative cell morphology of SH-SY5Y and viability assay after treatment with different concentrations of PrP^106-126^ (0, 100, and 200 µM) using the Cell Counting Kit-8 (CCK8 assay). All values represent means ± SD (n = 3, **p < 0.01. Statistical testing was done by One-way-ANOVA with *post hoc* Tukey’s multiple comparisons test. **(B)** Representative cell morphology of SH-SY5Y and viability assay after treatment with different concentrations of SW02 (1, 5, 10, and 20 µM) using CCK8 assay. All values represent means ± SD (n = 3). Statistical analysis was done by One-way-ANOVA with *post hoc* Tukey’s multiple comparisons test.

The amino acid sequence of PrP^106-126^ shares several physicochemical and biological properties with PrP^Sc^, including neurotoxicity. The viability of SH-SY5Y cells treated with different concentrations of PrP^106-126^ and SW02 for 24 h was evaluated using the CCK-8 assay. To confirm the neurotoxicity of the PrP^106-126^ peptide, SH-SY5Y cells treated with 100 and 200 μM PrP^106-126^ showed a significant decrease in viability and proliferation rate (*p* < 0.01; Figure 1A). Significant difference was not observed between 100 and 200 μM PrP^106-126^; therefore, 100 μM was selected as the optimal concentration to study the effects of PrP^106-126^ on the SH-SY5Y cells. Regarding SW02 exposure (1, 5, 10, and 20 μM), cell viability remained stable, with no significant cytotoxicity observed up to 20 μM (Figure 1B). The morphological appearance of cells treated with SW02 at 20 μM was similar to the non-exposed control (Figure 1B).

Consequently, the concentration of 20 μM SW02 was selected for subsequent experiments to elucidate the protective effects of SW02 against PrP^106-126^-induced neurotoxicity. These results indicated that SW02 maintained cell viability and PrP^106-126^-induced cell toxicity by inhibiting cell viability.

### 3.2 SW02 upregulates the gene expression of Hsp70 and Hsp70 ATPase activity

**FIGURE 2:**
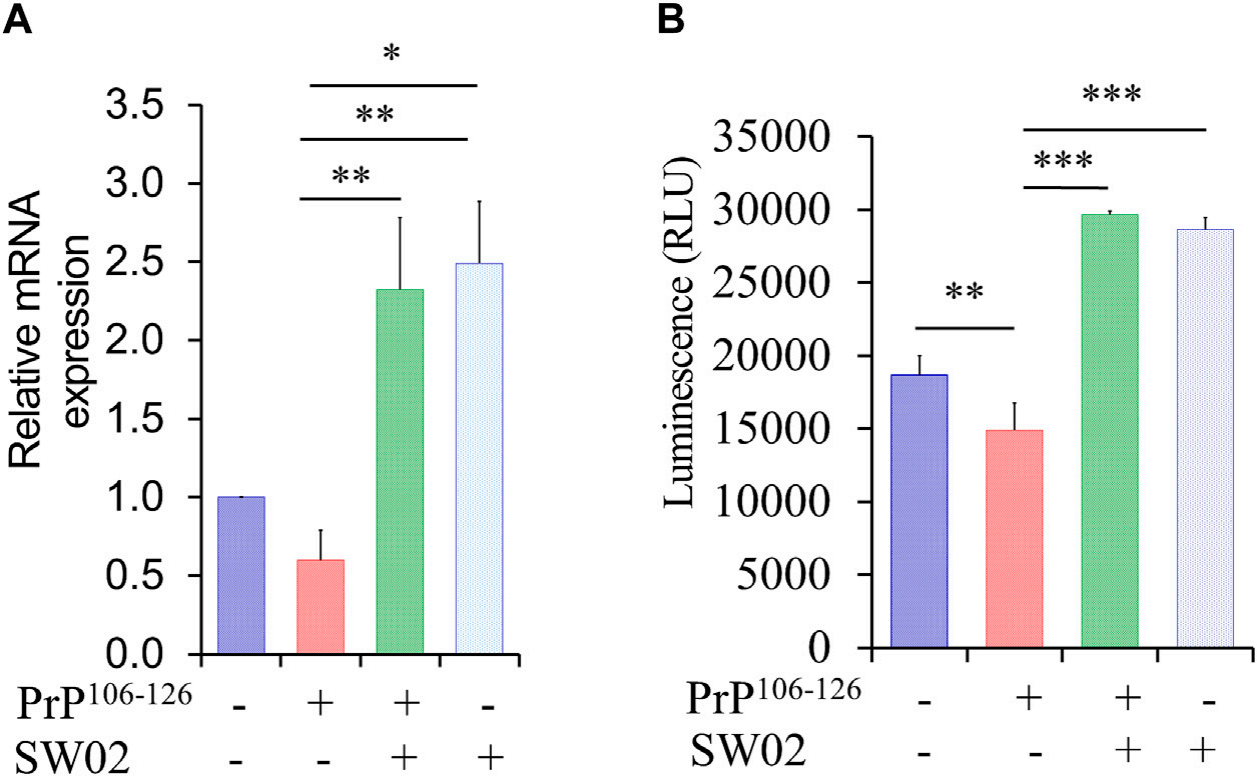
SW02 upregulated mRNA and ATPase activity of Hsp70. **(A)** The relative expression levels of Hsp70 in the treated cells. The data are reported as the mean ± SD; *p < 0.05, **p < 0.01. **(B)** SW02 upregulated heat shock protein 70 ATPase activity in SH-SY5Y cells however, PrP^106-126^ only downregulated ATPase activity. ATP in the cell lysate was detected via a luminescence assay. All values represent means ± SD (n = 3, *p < 0.05, **p < 0.01, and ***p < 0.001). Statistical analysis was done by One-way-ANOVA with *post hoc* Tukey’s multiple comparisons test.

Hsp70 and its ATPase activities are known to be responsible for the regulation of various biological processes. A representative function of the Hsp70 family is chaperone activity such as protein folding, suppression of protein aggregation, removal of misfolded proteins, and regulation of assembly/disassembly of protein complexes (Bukau and Horwich, 1998; Cho et al., 2015). To determine whether SW02 upregulates Hsp70 and Hsp70 ATPase activity, SH-SY5Y cells were pretreated with SW02 followed by the PrP^106-126^ peptide. As shown in Figure 2A, treatment with SW02 significantly upregulated Hsp70 mRNA expression in SH-SY5Y cells (*p* < 0.01). Of note, treatment with SW02 alone was sufficient to enhance Hsp70 expression.

On the other hand, a higher amount of ATPase was detected in the cell lysate of SH-SY5Y cells treated with SW02 (*p* < 0.001; Figure 2B). In addition, the ATPase amount was higher in cells treated with SW02 only. In contrast, the release of ATPase significantly decreased when cells were treated with the PrP^106-126^ peptide (*p* < 0.01; Figure 2B).

### 3.3 SW02 attenuates PrP^106-126^-induced neurotoxicity

**FIGURE 3:**
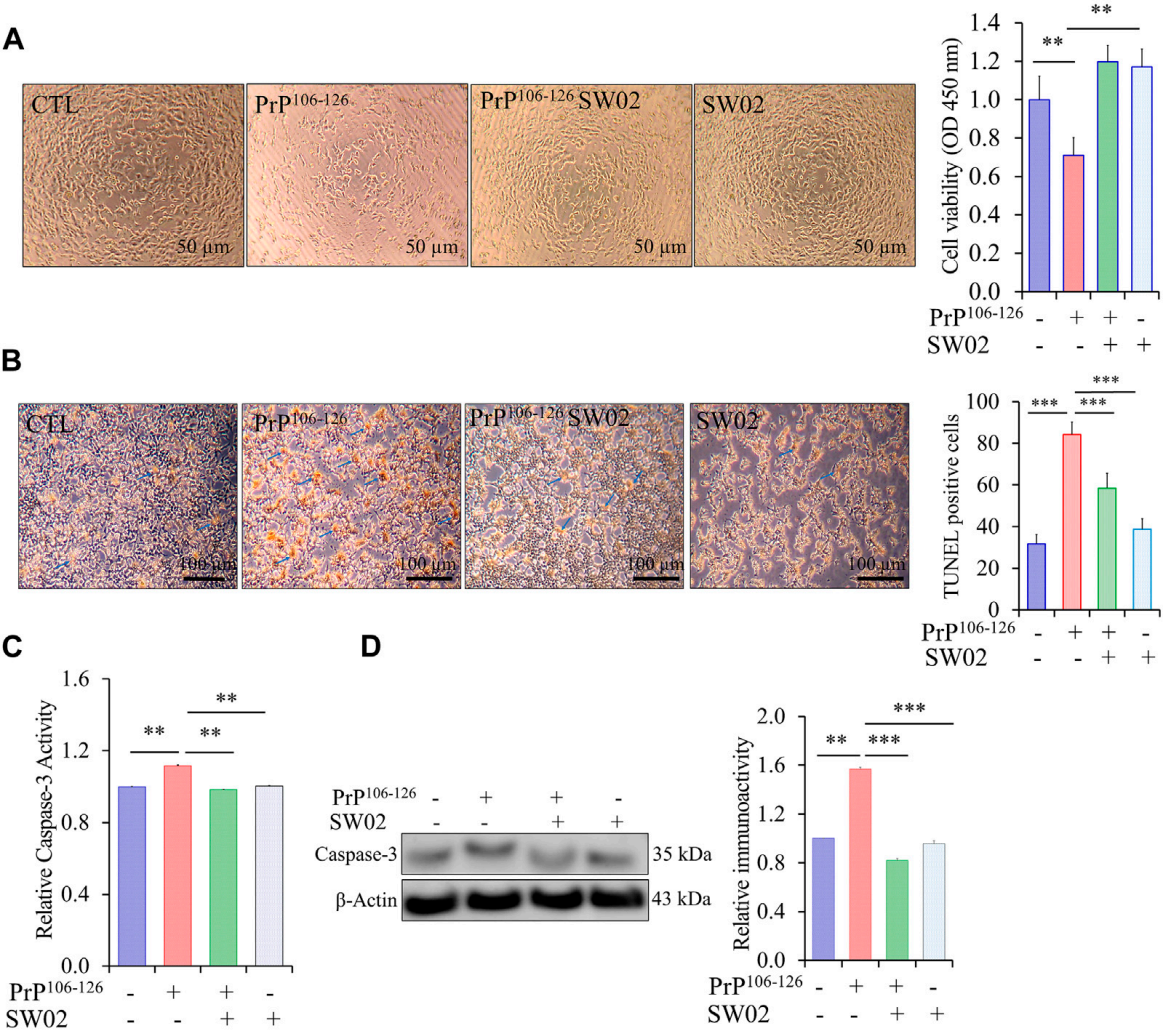
SW02 (20 µM) enhanced SH-SY5Y cell viability and inhibited apoptosis induced by PrP^106-126^ (100 µM). **(A)** SW02 increased the cell viability compared with the PrP^106-126^ positive control. The images of representative cell morphology are from one of three separate experiments. Statistical analysis was done by One-way-ANOVA with *post hoc* Tukey’s multiple comparisons test. Error bars represent SD (**p < 0.01). **(B)** SW02 protected against PrP^106-126^-induced cell apoptosis. SH-SY5Y cell apoptosis was assayed using Terminal deoxynucleotidyl transferase dUTP end labeling staining. The brown substrate at the site of DNA fragmentation indicates apoptotic cell nuclei. The images of representative cell morphology are from one of three separate experiments. **(C)** The Caspase-3 Assay Kit (Colorimetric) shows the rescue effect of SW02 against PrP^106-126^-induced cell apoptosis based on caspase-3 activity. **(D)** Expression and quantitative analysis of caspase-3 were performed using Western blot analysis *p < 0.05. All values represent means ± SD (n = 3, **p < 0.01). Statistical analysis was done by One-way-ANOVA with *post hoc* Tukey’s multiple comparisons test.

To assess the protective effects of SW02 against PrP^106-126^-induced neurotoxicity, SH-SY5Y cells were pretreated with SW02 before the addition of the PrP^106-126^ peptide. SW02 exposure protected the cells from PrP^106-126^-induced viability inhibition. Cell viability measured using the CCK8 assay was significantly increased by SW02 compared with the PrP^106-126^ positive control (*p* < 0.01; Figure 3A).

To further investigate whether SW02 prevents apoptotic cell death in PrP^106-126^-induced cytotoxicity, TUNEL staining was used to identify cells undergoing apoptosis. PrP^106-126^ peptide treatment significantly increased apoptotic cells (dark brown; Figure 3B). Notably, the TUNEL assay showed that SW02 treatment inhibited SH-SY5Y cell apoptosis induced by the PrP^106-126^ peptide compared with the PrP^106-126^ control (Figure 3B). SW02 also markedly prevented the activation of caspase-3, a pro-apoptotic enzyme (Figure 3C). Notably, cells treated with SW02 only did not activate caspase-3. Conversely, caspase-3 was activated in PrP^106-126^-treated cells based on results from the caspase-3 activity assay kit (*p* < 0.01; Figure 3C). To confirm the result of the caspase-3 activity, the protein level of caspase-3 was further examined by Western blot analysis. The findings indicated that SW02 significantly decreased the protein level of caspase-3 which was increased upon treatment with PrP^106-126^ (Figure 3D).

Consequently, these findings indicated that SW02 protects against the PrP^106-126^ peptide by inhibiting apoptotic cell production and preserving cell viability in PrP^106-126^-treated SH-SY5Y cells.

### 3.4 SW02 increases neurite outgrowth activity

**FIGURE 4:**
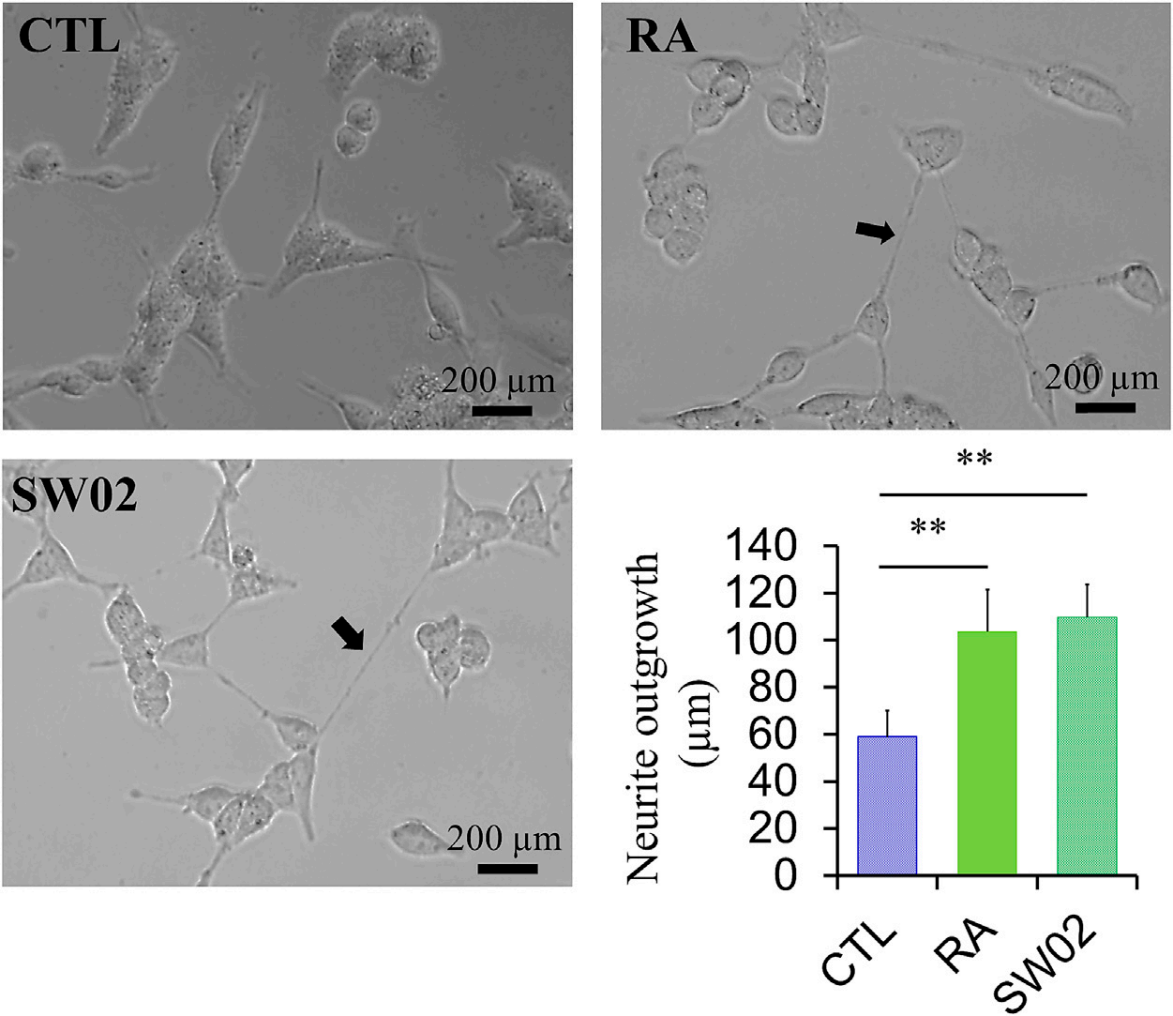
Representative micrographs were used to quantify neurite extension in SH-SY5Y cells after incubation with SW02 (20 µM). SW02 increased the neurite extension of SH-SY5Y cells. All values represent means ± SD (n = 3, **p < 0.01). Retinoic acid (RA) was used as a positive control to induce neurite outgrowth. Statistical analysis was done by One-way-ANOVA with *post hoc* Tukey’s multiple comparisons test.

Neurite outgrowth is a fundamental process in the formation of neural networks and nerve regeneration (Sherman and Bang, 2018). Following injury, neurite outgrowth plays a key role in the regeneration of the nervous system. To evaluate the effects of SW02 on neurite formation, cells were treated with SW02. SW02 significantly increased neurite extension in SH-SY5Y cells (*p* < 0.01; Figure 4). The results suggest a potential neuroregenerative effect of SW02.

### 3.5 The therapeutic effects of SW02 in ME7 scrapie-infected mice

**FIGURE 5:**
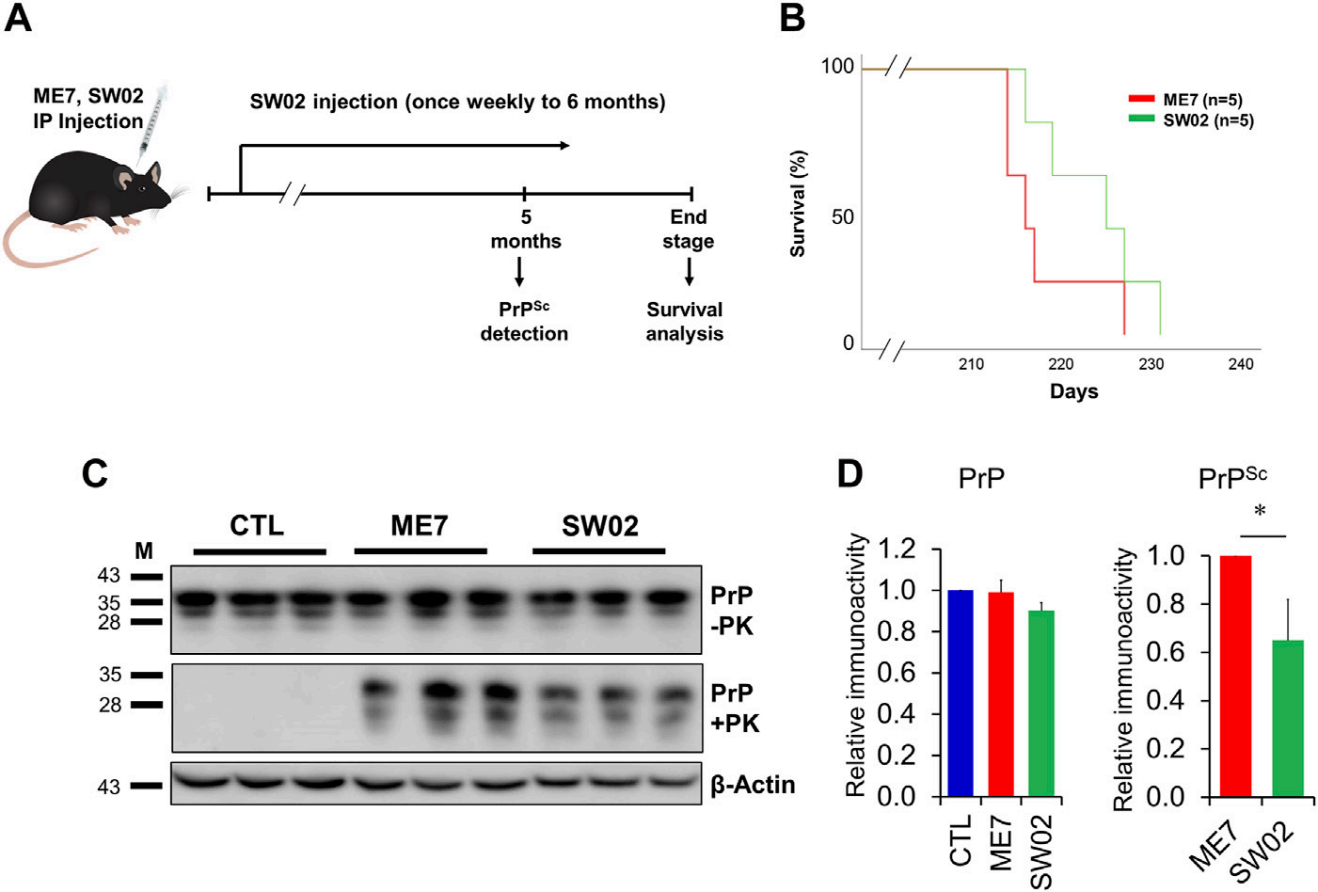
Evaluation of the protective effects of SW02 in ME7-infected mice. **(A)** Experimental outline of the assessment of the effects of heat shock protein 70 activator, SW02 (20 µM), in ME7 scrapie-infected mice. **(B)** Kaplan-Meier survival curve was generated to show the effects of SW02 on the survival of ME7-infected mice (n = 5 individual animals). **(C)** Evaluation of the protective effects of SW02 on PrP and PrP^Sc^ accumulation expression in the ME7 scrapie-infected mice at 5 months post-injection based on western blotting. β-actin was used as an internal control. **(D)** The quantitative analyses of PrP and PrP^Sc^ immunoblot *p < 0.05. All data are expressed as the means ± standard deviation (n = 3). Student’s t-test and one-way ANOVA were used for comparison between two groups and multiple groups, respectively.

Several studies have demonstrated that Hsp70 plays key roles in neuroprotection through different processes (Venediktov et al., 2023). Therefore, small molecule-based activators of Hsp70 have been exploited for use as potential therapeutic agents. To assess the therapeutic effects of SW02 against prion disease, the ME7 scrapie strain was inoculated into 6-week-old mice, followed by weekly treatment with or without SW02. The mice were sacrificed at two-time points, 5 months and at the terminal stage post-injection (Figure 5A). Although a significant difference was not found in the survival analysis between the ME7 scrapie-infected untreated mice (control) and SW02-treated ME7 scrapie-infected mice (*p* = 0.16), the survival time was longer in the SW02-treated mice (223.6 ± days) compared with the untreated control mice (217.6 ± 5.4 days; Figure 5B).

Brain tissues from mice were homogenized and analyzed using western blot to detect total PrP and PrP^Sc^. The total PrP expression in the SW02-treated mice was similar to the untreated mice at 5 months (*p* = 0.42; Figure 5C, D). PrP^Sc^ accumulation in SW02-treated ME7 scrapie-infected mice was significantly decreased at 5 months compared with untreated ME7 scrapie-infected mice (*p* < 0.05; Figure 5C, D). At the terminal stage of the disease, however, a difference was not observed in PrP^Sc^ levels between the SW02-treated and untreated groups (Supplementary Figure S2).

### 3.6 SW02 treatment does not decrease reactive astrocytes

**FIGURE 6:**
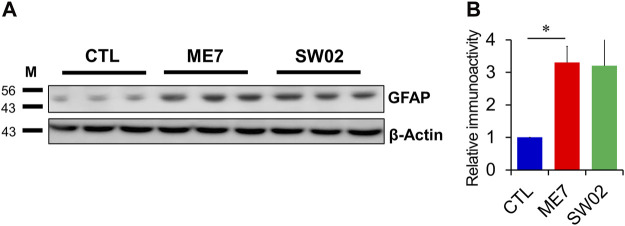
Evaluation of astrocyte in the ME7 scrapie-infected mice by western blot analysis. **(A)** Protein expression of glial fibrillary acidic protein (GFAP), astrocyte marker, in the ME7 scrapie-infected mice at 5 months post-injection based on western blotting. β-actin was used as an internal control. **(B)** The quantitative analyses of GFAP immunoblot. The results in the graph are the mean ± SD. All data are expressed as the means ± SD (n = 3, **p < 0.05). Statistical analysis was done by One-way-ANOVA with *post hoc* Tukey’s multiple comparisons test.

GFAP was used to detect astrocytes reactivity throughout the brain in response to most forms of central nervous system damage. The results showed a non-significant difference in GFAP expression at 5 months (*p* =  0.47; Figure 6A, B) in the SW02-treated ME7 scrapie-infected mice compared with the untreated ME7 scrapie-infected mice. However, GFAP was significantly higher expressed (*p* < 0.05) in the ME7 scrapie-infected mice compared with the negative control mice (Figure 6A, B), indicating an upregulation of neuroinflammation.

## 4 Discussion

In the present study, the effects of SW02 as a potential therapeutic agent against prion disease were investigated. First, the protective effects of SW02 against PrP^106-126^-induced neurotoxicity were evaluated using SH-SY5Y cells followed by assessment of its therapeutic effects in prion-infected mice. The results provided evidence that SW02 protected PrP^106-126^-treated SH-SY5Y cells from neurotoxicity. However, *in vivo*, although SW02 did not show a significant difference in survival time, PrP^Sc^ accumulation at 5 months post-infection decreased. In the current study, SW02, a stimulator of Hsp70 ATPase activity, was utilized (Chang et al., 2008). SW02 was confirmed to stimulate Hsp70 ATPase activity; SW02-treated SH-SY5Y cells increased the Hsp70 ATPase activity, indicating the SW02 mechanism may function via ATPase activation of Hsp70 (Hu et al., 2022; Rutledge et al., 2022).

SH-SY5Y is a cell-based model for different neurodegenerative diseases (Xicoy et al., 2017), including prion diseases (O’donovan et al., 2001; Puig et al., 2016). Accordingly, in the present study, SH-SY5Y was used as an *in vitro* cell model for prion diseases to study cell viability, apoptosis, and neurite extension. In addition, because PrP^106-126^ induces neuronal apoptosis and cytotoxicity, it is commonly used as a model for examining PrP^Sc^ neurotoxicity (O’donovan et al., 2001; Seo et al., 2010; Ning and Mu, 2018). The results of this study consistently showed the PrP^106-126^ peptide decreased cell viability and increased apoptotic cells based on the TUNEL assay. In addition, caspase-3, which participates in the cell apoptosis process and DNA fragmentation (Brentnall et al., 2013), was significantly upregulated, indicating PrP^106-126^ is an efficient model for *in vitro* study of prion-induced cell death and maintains the pathological properties of misfolded prions. However, SW02 alleviated the cell viability in PrP^106-126^-treated SH-SY5Y cells. Furthermore, SW02 inhibited cell apoptosis by decreasing the apoptotic cell death, caspase-3 activity, and the protein level of caspase-3, indicating a suppressive effect on the apoptosis process (Ko et al., 2015). The observed effects of SW02 are likely attributed to the biological activities of the stimulated Hsp70 ATPase activity (Kwong et al., 2015; Hu et al., 2022). To study whether the SW02 increases the Hsp70 expression, we performed RT-PCR analysis and observed a higher expression of Hsp70 mRNA in cells treated with SW02. Similarly, Lui and Kong showed that Hsp70 exerts an anti-apoptotic activity (Lui and Kong, 2007). In addition, Hsp70 participated in rescuing cells from apoptosis more than other Hsps (Vasaikar et al., 2015). It has been shown that the ATPase activity of SW02 enhances Hsp70 function and targeting the ATPase activity of Hsp70 regulates tau accumulation (Jinwal et al., 2009). In the same study, the authors investigated whether Hsp70 and Hsp90 inhibitors could facilitate the clearance of tau mutants. They found that while Hsp90 inhibitors showed varied effects, Hsp70 inhibition significantly reduced tau levels. These findings highlight a distinction in the mechanism of action between Hsp90 and Hsp70 inhibitors. Together, these findings are consistent with previous studies indicating stimulation of the Hsp70 ATPase activity attenuates neurotoxicity (Pratt and Toft, 2003; Kumar et al., 2007).

Neurite outgrowth motivates the connecting of the nervous system during development and regeneration after disease. In the present study, SH-SY5Y cells treated with SW02 were shown to have increased neurite outgrowth, indicating SW02 may contribute to the regeneration of damaged neurons induced by prion disease. However, further experiments are required to show the contribution of SW02 in neuroregeneration process. Hsp70 chaperone and their stimulators were previously shown to promote neurite outgrowth (Takeuchi et al., 2002; Evans et al., 2010; Lackie et al., 2017).

Drugs known to induce Hsp70 expression, such as 17-DMAG and dexamethasone, have been explored for their therapeutic potential in treating prion diseases in *Drosophila*. However, significant outcomes were not observed when the individual treatment was used; however, their combination significantly increased the level of inducible Hsp70, decreased PrP expression, inhibited the accumulation of PrP^Sc^, and enhanced locomotor activity (Zhang et al., 2014). Recently, Thackray et al. showed that metazoan Hsp70 disaggregase system protects neurons from prion toxicity and reduces the prion seeding activity in scrapie prion-exposed ovine PrP *Drosophila* (Thackray et al., 2022). In the present study, the survival time was longer in the SW02-treated group than in the control group but without statistical significance. In addition, western blot analysis revealed a lower PrP^Sc^ level in the SW02-treated group after 5 months compared with the control group. However, a possible explanation for the lack of effect on increasing the survival time could be due to a partial functional activity of SW02 against prion disease. Therefore, a combination action of two pharmacological activators of Hsp70 may maximize the effectiveness of each intervention, as reported by Zhang et al. (2014). Higher GFAP levels have been reported to correlate with prion disease in brain tissue (Lakkaraju et al., 2022). In the present study, SW02 treatment did not decrease GFAP expression compared with the untreated mice and was likely due to the high PrP^Sc^ levels. Another important aspect that may contribute to the lack of decreased GFAP levels is the association with the immunostimulatory properties of Hsp70 (Borges et al., 2012).

We acknowledge that our study has certain limitations, and further work is needed to elucidate the details of the mechanism of SW02 action. In addition, using different neuronal cell types to investigate the potential protective effects of SW02 is required.

To the best of our knowledge, Hsp70 activators have not received consideration in the field of neurodegenerative diseases. Because cures or palliative therapies for prion diseases do not currently exist, the results of the present study showing the neuroprotective potential of SW02 are promising. This is the first study in which the therapeutic effects of SW02 against prion disease were shown. Furthermore, additional research studies in which the therapeutic potential of SW02 in other scrapie strain-infected mice models (RML, 22L, 137A, and Chandler) are investigated are required. Reportedly, other Hsps have a protective effect in animal models of prion disease (Jackrel and Shorter, 2017). Thus, studies in which other Hsps are used are needed to investigate their therapeutic effects against prion disease.

## 5 Conclusion

The findings showed that SW02 has neuroprotective properties against PrP^106-126^-induced neurotoxicity. *In vitro* results indicated that SW02 reduces neurotoxicity-mediated apoptosis by downregulating cell death and caspase-3 activity. SW02 also exhibited enhanced neurite extension and Hsp70 ATPase activity. Although prion-infected mice treated with SW02 showed non-significant results compared with the control mice, the SW02-treated mice exhibited a longer survival time. Further research is required to determine the precise mechanisms through which SW02 exerts neuroprotective effects in other scrapie strain-infected mice such as RML, 22L, 137A, and Chandler.

## Data Availability

The original contributions presented in the study are included in the article/Supplementary Material, further inquiries can be directed to the corresponding author.

## References

[B1] Beretta G. Shala A. L. (2022). Impact of heat shock proteins in neurodegeneration: possible therapeutical targets. Ann. Neurosci. 29, 71–82. 10.1177/09727531211070528 35875428 PMC9305912

[B2] Borges T. Wieten L. Van Herwijnen M. Broere F. Van Der Zee R. Bonorino C. (2012). The anti-inflammatory mechanisms of hsp70. Front. Immunol. 3, 95. 10.3389/fimmu.2012.00095 22566973 PMC3343630

[B3] Brentnall M. Rodriguez-Menocal L. De Guevara R. L. Cepero E. Boise L. H. (2013). Caspase-9, caspase-3 and caspase-7 have distinct roles during intrinsic apoptosis. BMC Cell Biol. 14, 32. 10.1186/1471-2121-14-32 23834359 PMC3710246

[B4] Bukau B. Horwich A. L. (1998). The hsp70 and hsp60 chaperone machines. Cell 92, 351–366. 10.1016/s0092-8674(00)80928-9 9476895

[B5] Caughey B. (2003). Prion protein conversions: insight into mechanisms, tse transmission barriers and strains. Br. Med. Bull. 66, 109–120. 10.1093/bmb/66.1.109 14522853

[B6] Chang L. Bertelsen E. B. Wisén S. Larsen E. M. Zuiderweg E. R. Gestwicki J. E. (2008). High-throughput screen for small molecules that modulate the atpase activity of the molecular chaperone dnak. Anal. Biochem. 372, 167–176. 10.1016/j.ab.2007.08.020 17904512

[B7] Chernova T. A. Wilkinson K. D. Chernoff Y. O. (2017). Prions, chaperones, and proteostasis in yeast. Cold Spring Harb. Perspect. Biol. 9, a023663. 10.1101/cshperspect.a023663 27815300 PMC5287078

[B8] Cho H. J. Kim G. H. Park S. H. Hyun J. Y. Kim N. K. Shin I. (2015). Probing the effect of an inhibitor of an atpase domain of hsc70 on clathrin-mediated endocytosis. Mol. Biosyst. 11, 2763–2769. 10.1039/c4mb00695j 25728281

[B9] Cummings C. J. Sun Y. Opal P. Antalffy B. Mestril R. Orr H. T. (2001). Over-expression of inducible hsp70 chaperone suppresses neuropathology and improves motor function in sca1 mice. Hum. Mol. Genet. 10, 1511–1518. 10.1093/hmg/10.14.1511 11448943

[B10] Dutta D. J. Hashimoto-Torii K. Torii M. (2021). “Role of heat shock factor 1 in neural development and disorders,” in Heat shock proteins in inflammatory diseases. Editors Asea A. A. A. Kaur P. (Cham: Springer International Publishing).

[B11] Evans C. G. Chang L. Gestwicki J. E. (2010). Heat shock protein 70 (hsp70) as an emerging drug target. J. Med. Chem. 53, 4585–4602. 10.1021/jm100054f 20334364 PMC2895966

[B12] Evans C. G. Wisén S. Gestwicki J. E. (2006). Heat shock proteins 70 and 90 inhibit early stages of amyloid beta-(1-42) aggregation *in vitro* . J. Biol. Chem. 281, 33182–33191. 10.1074/jbc.M606192200 16973602

[B13] Glatzel M. Sigurdson C. J. (2019). Recent advances on the molecular pathogenesis of prion diseases. Brain Pathol. 29, 245–247. 10.1111/bpa.12693 30588674 PMC8028283

[B14] Halliday M. Radford H. Mallucci G. R. (2014). Prions: generation and spread versus neurotoxicity. J. Biol. Chem. 289, 19862–19868. 10.1074/jbc.R114.568477 24860100 PMC4106307

[B15] Han H. J. Kim S. Kwon J. (2019). Thymosin beta 4-induced autophagy increases cholinergic signaling in prp (106-126)-treated ht22 cells. Neurotox. Res. 36, 58–65. 10.1007/s12640-018-9985-0 30552633

[B16] Hu C. Yang J. Qi Z. Wu H. Wang B. Zou F. (2022). Heat shock proteins: biological functions, pathological roles, and therapeutic opportunities. MedComm 3, e161. 10.1002/mco2.161 35928554 PMC9345296

[B17] Jackrel M. E. Shorter J. (2017). Protein-remodeling factors as potential therapeutics for neurodegenerative disease. Front. Neurosci. 11, 99. 10.3389/fnins.2017.00099 28293166 PMC5328956

[B18] Jinwal U. K. Koren J. O’leary J. C. Jones J. R. Abisambra J. F. Dickey C. A. (2010). Hsp70 atpase modulators as therapeutics for alzheimer’s and other neurodegenerative diseases. Mol. Cell Pharmacol. 2, 43–46. 20523917 PMC2879647

[B19] Jinwal U. K. Miyata Y. Koren J. Jones J. R. Trotter J. H. Chang L. (2009). Chemical manipulation of hsp70 atpase activity regulates tau stability. J. Neurosci. 29, 12079–12088. 10.1523/JNEUROSCI.3345-09.2009 19793966 PMC2775811

[B20] Kenward N. Hope J. Landon M. Mayer R. J. (1994). Expression of polyubiquitin and heat-shock protein 70 genes increases in the later stages of disease progression in scrapie-infected mouse brain. J. Neurochem. 62, 1870–1877. 10.1046/j.1471-4159.1994.62051870.x 7512619

[B21] Kim Y. C. Won S. Y. Jeong B. H. (2021). Altered expression of glymphatic system-related proteins in prion diseases: implications for the role of the glymphatic system in prion diseases. Cell Mol. Immunol. 18, 2281–2283. 10.1038/s41423-021-00747-z 34363029 PMC8429425

[B22] Ko S.-K. Kim J. Na D. c. Park S. Park S.-H. Hyun Ji y. (2015). A small molecule inhibitor of atpase activity of hsp70 induces apoptosis and has antitumor activities. Chem. Biol. 22, 391–403. 10.1016/j.chembiol.2015.02.004 25772468

[B23] Kovacs G. G. Budka H. (2009). Molecular pathology of human prion diseases. Int. J. Mol. Sci. 10, 976–999. 10.3390/ijms10030976 19399233 PMC2672014

[B24] Kovács G. G. Kurucz I. Budka H. Adori C. Müller F. Acs P. (2001). Prominent stress response of purkinje cells in creutzfeldt-jakob disease. Neurobiol. Dis. 8, 881–889. 10.1006/nbdi.2001.0418 11592855

[B25] Krance S. H. Luke R. Shenouda M. Israwi A. R. Colpitts S. J. Darwish L. (2020). Cellular models for discovering prion disease therapeutics: progress and challenges. J. Neurochem. 153, 150–172. 10.1111/jnc.14956 31943194

[B26] Kumar P. Ambasta R. K. Veereshwarayya V. Rosen K. M. Kosik K. S. Band H. (2007). Chip and hsps interact with beta-app in a proteasome-dependent manner and influence abeta metabolism. Hum. Mol. Genet. 16, 848–864. 10.1093/hmg/ddm030 17317785

[B27] Kwong J. M. Gu L. Nassiri N. Bekerman V. Kumar-Singh R. Rhee K. D. (2015). Aav-mediated and pharmacological induction of hsp70 expression stimulates survival of retinal ganglion cells following axonal injury. Gene Ther. 22, 138–145. 10.1038/gt.2014.105 25427613 PMC4320032

[B28] Lackie R. E. Maciejewski A. Ostapchenko V. G. Marques-Lopes J. Choy W. Y. Duennwald M. L. (2017). The hsp70/hsp90 chaperone machinery in neurodegenerative diseases. Front. Neurosci. 11, 254. 10.3389/fnins.2017.00254 28559789 PMC5433227

[B29] Lakkaraju A. K. K. Sorce S. Senatore A. Nuvolone M. Guo J. Schwarz P. (2022). Glial activation in prion diseases is selectively triggered by neuronal prp(sc). Brain Pathol. 32, e13056. 10.1111/bpa.13056 35178783 PMC9425016

[B30] Li C. Wang D. Wu W. Yang W. Ali Shah S. Z. Zhao Y. (2018). Dlp1-dependent mitochondrial fragmentation and redistribution mediate prion-associated mitochondrial dysfunction and neuronal death. Aging Cell 17, e12693. 10.1111/acel.12693 29166700 PMC5771399

[B31] Liberski P. P. Ironside J. W. (2015). “Chapter 23 - prion diseases,” in Neurobiology of brain disorders. Editors Zigmond M. J. Rowland L. P. Coyle J. T. (San Diego: Academic Press).

[B32] Lui J. C. Kong S. K. (2007). Heat shock protein 70 inhibits the nuclear import of apoptosis-inducing factor to avoid DNA fragmentation in tf-1 cells during erythropoiesis. FEBS Lett. 581, 109–117. 10.1016/j.febslet.2006.11.082 17182042

[B33] Marber M. S. Mestril R. Chi S. H. Sayen M. R. Yellon D. M. Dillmann W. H. (1995). Overexpression of the rat inducible 70-kd heat stress protein in a transgenic mouse increases the resistance of the heart to ischemic injury. J. Clin. Invest 95, 1446–1456. 10.1172/JCI117815 7706448 PMC295626

[B34] Mayer M. P. Bukau B. (2005). Hsp70 chaperones: cellular functions and molecular mechanism. Cell Mol. Life Sci. 62, 670–684. 10.1007/s00018-004-4464-6 15770419 PMC2773841

[B35] Mays C. E. Armijo E. Morales R. Kramm C. Flores A. Tiwari A. (2019). Prion disease is accelerated in mice lacking stress-induced heat shock protein 70 (hsp70). J. Biol. Chem. 294, 13619–13628. 10.1074/jbc.RA118.006186 31320473 PMC6746463

[B36] Miller D. J. Fort P. E. (2018). Heat shock proteins regulatory role in neurodevelopment. Front. Neurosci. 12, 821. 10.3389/fnins.2018.00821 30483047 PMC6244093

[B37] Moore R. A. Taubner L. M. Priola S. A. (2009). Prion protein misfolding and disease. Curr. Opin. Struct. Biol. 19, 14–22. 10.1016/j.sbi.2008.12.007 19157856 PMC2674794

[B38] Ning L. Mu Y. (2018). Aggregation of prp106–126 on surfaces of neutral and negatively charged membranes studied by molecular dynamics simulations. Biochimica Biophysica Acta (BBA) - Biomembr. 1860, 1936–1948. 10.1016/j.bbamem.2018.03.009 29550288

[B39] O’donovan C. N. Tobin D. Cotter T. G. (2001). Prion protein fragment prp-(106–126) induces apoptosis via mitochondrial disruption in human neuronal sh-sy5y cells. J. Biol. Chem. 276, 43516–43523. 10.1074/jbc.M103894200 11533027

[B40] Park Y. G. Jeong J. K. Moon M. H. Lee J. H. Lee Y. J. Seol J. W. (2012). Insulin-like growth factor-1 protects against prion peptide-induced cell death in neuronal cells via inhibition of bax translocation. Int. J. Mol. Med. 30, 1069–1074. 10.3892/ijmm.2012.1087 22895829

[B41] Petrucelli L. Dickson D. Kehoe K. Taylor J. Snyder H. Grover A. (2004). Chip and hsp70 regulate tau ubiquitination, degradation and aggregation. Hum. Mol. Genet. 13, 703–714. 10.1093/hmg/ddh083 14962978

[B42] Pratt W. B. Toft D. O. (2003). Regulation of signaling protein function and trafficking by the hsp90/hsp70-based chaperone machinery. Exp. Biol. Med. (Maywood) 228, 111–133. 10.1177/153537020322800201 12563018

[B43] Puig B. Altmeppen H. C. Ulbrich S. Linsenmeier L. Krasemann S. Chakroun K. (2016). Secretory pathway retention of mutant prion protein induces p38-mapk activation and lethal disease in mice. Sci. Rep. 6, 24970. 10.1038/srep24970 27117504 PMC4847012

[B44] Ritchie D. L. Ironside J. W. (2017). “Chapter fourteen - neuropathology of human prion diseases,” in Progress in molecular biology and translational science. Editors Legname G. Vanni S. (Academic Press). 10.1016/bs.pmbts.2017.06.011 28838666

[B45] Rosenzweig R. Nillegoda N. B. Mayer M. P. Bukau B. (2019). The hsp70 chaperone network. Nat. Rev. Mol. Cell Biol. 20, 665–680. 10.1038/s41580-019-0133-3 31253954

[B46] Rutledge B. S. Choy W.-Y. Duennwald M. L. (2022). Folding or holding?hsp70 and hsp90 chaperoning of misfolded proteins in neurodegenerative disease. J. Biol. Chem. 298, 101905. 10.1016/j.jbc.2022.101905 35398094 PMC9079180

[B47] Santana E. De Los Reyes T. Casas-Tintó S. (2020). Small heat shock proteins determine synapse number and neuronal activity during development. PLOS ONE 15, e0233231. 10.1371/journal.pone.0233231 32437379 PMC7241713

[B48] Scheckel C. Aguzzi A. (2018). Prions, prionoids and protein misfolding disorders. Nat. Rev. Genet. 19, 405–418. 10.1038/s41576-018-0011-4 29713012

[B49] Seo J. S. Seol J. W. Moon M. H. Jeong J. K. Lee Y. J. Park S. Y. (2010). Hypoxia protects neuronal cells from human prion protein fragment-induced apoptosis. J. Neurochem. 112, 715–722. 10.1111/j.1471-4159.2009.06496.x 19919574

[B50] Sherman S. P. Bang A. G. (2018). High-throughput screen for compounds that modulate neurite growth of human induced pluripotent stem cell-derived neurons. Dis. Model Mech. 11, dmm031906. 10.1242/dmm.031906 29361516 PMC5894944

[B51] Takeuchi H. Kobayashi Y. Yoshihara T. Niwa J. Doyu M. Ohtsuka K. (2002). Hsp70 and hsp40 improve neurite outgrowth and suppress intracytoplasmic aggregate formation in cultured neuronal cells expressing mutant sod1. Brain Res. 949, 11–22. 10.1016/s0006-8993(02)02568-4 12213295

[B52] Thackray A. M. Lam B. Mcnulty E. E. Nalls A. V. Mathiason C. K. Magadi S. S. (2022). Clearance of variant creutzfeldt-jakob disease prions *in vivo* by the hsp70 disaggregase system. Brain 145, 3236–3249. 10.1093/brain/awac144 35446941 PMC9473358

[B53] Vabulas R. M. Raychaudhuri S. Hayer-Hartl M. Hartl F. U. (2010). Protein folding in the cytoplasm and the heat shock response. Cold Spring Harb. Perspect. Biol. 2, a004390. 10.1101/cshperspect.a004390 21123396 PMC2982175

[B54] Vasaikar S. Ghosh S. Narain P. Basu A. Gomes J. (2015). Hsp70 mediates survival in apoptotic cells—boolean network prediction and experimental validation. Front. Cell. Neurosci. 9, 319. 10.3389/fncel.2015.00319 26379495 PMC4548197

[B55] Venediktov A. A. Bushueva O. Y. Kudryavtseva V. A. Kuzmin E. A. Moiseeva A. V. Baldycheva A. (2023). Closest horizons of hsp70 engagement to manage neurodegeneration. Front. Mol. Neurosci. 16, 1230436. 10.3389/fnmol.2023.1230436 37795273 PMC10546621

[B56] Xicoy H. Wieringa B. Martens G. J. M. (2017). The sh-sy5y cell line in Parkinson’s disease research: a systematic review. Mol. Neurodegener. 12, 10. 10.1186/s13024-017-0149-0 28118852 PMC5259880

[B57] Zayed M. Kook S.-H. Jeong B.-H. (2023). Potential therapeutic use of stem cells for prion diseases. Cells 12, 2413. 10.3390/cells12192413 37830627 PMC10571911

[B58] Zhang Y. Casas-Tinto S. Rincon-Limas D. E. Fernandez-Funez P. (2014). Combined pharmacological induction of hsp70 suppresses prion protein neurotoxicity in drosophila. PLos One 9, e88522. 10.1371/journal.pone.0088522 24523910 PMC3921213

