# Supplementary material for: Assessment of the therapeutic potential of Hsp70 activator against prion diseases using in vitro and in vivo models
Source: Front Cell Dev Biol. 2024 Jul 22;12:1411529. doi: 10.3389/fcell.2024.1411529 (PMC11298377; doi:10.3389/fcell.2024.1411529)
Supplement: Supplementary file 1 [file DataSheet1.docx]

Supplementary Material


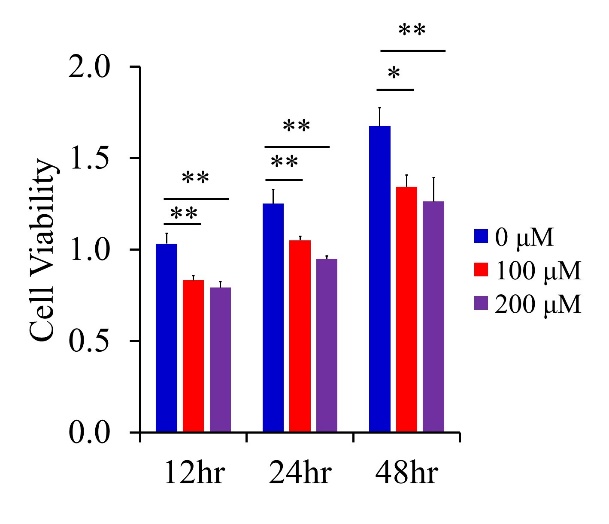


**Supplementary Figure 1**. Longer exposure of SH-SY5Y cell line to PrP^106-126^. The proliferation rate was assessed at different time points; 12, 24, and 48 hr using CCK8 assay. The results showed that cells exposed to PrP^106-126^ (100 and 200 μM) have similar effects at 12, 24, and 48 hrs. Thus, 24 hr time point treatment has been selected.


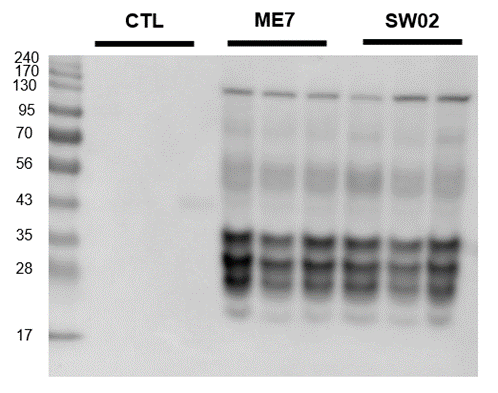


**Supplementary Figure 2.** Evaluation of the protective effects of SW02 on PrP^Sc^ accumulation expression in the ME7 scrapie-infected mice at the terminal stage based on western blotting.
